# Flexible and non-invasive passive integrated transponder (PIT) tagging protocols for tropical freshwater fish species

**DOI:** 10.1016/j.mex.2018.04.001

**Published:** 2018-04-06

**Authors:** Bettina Grieve, J. Baumgartner Lee, Wayne Robinson, Luiz G.M. Silva, Karl Pomorin, Garry Thorncraft, Nathan Ning

**Affiliations:** a Institute of Land, Water and Society, Charles Sturt University, P.O. Box 789, Albury, NSW, 2640, Australia; b Karltek Pty Ltd., Sanctuary Lakes, Melbourne, VIC, Australia; c National University of Laos, Dongdok Campus, Vientiane, Lao People’s Democratic Republic; d Universidade Federal de São João del-Rei, PPGTDS, PGE, Campus Alto Paraopeba, MG, Brazil

**Keywords:** Flexible passive integrated transponder, PIT tagging protocols for tropical freshwater species, Mark-recapture techniques, Striped catfish, Goldfin tinfoil barbs, Mekong River

## Abstract

Passive integrated transponder (PIT) tagging has proven to be an effective mark-recapture technique for many temperate freshwater and marine fish species, but its adaptability to tropical freshwater species remains largely unknown. Nevertheless, many tropical river systems, such as the Mekong in South East Asia, are currently being developed at an unprecedented rate for their relatively abundant water resources. Consequently, there is an urgent need for efficient mark-recapture technologies to understand and assess the impacts of human developments on the movement ecology of tropical freshwater fish species. This paper discusses the development of an optimal protocol for PIT tagging tropical freshwater fishes, using two Mekong River species – Striped catfish (*Pangasianodon hypophthalmus*) and Goldfin tinfoil barb (*Hypsibarbus malcolmi*) – as model species.

• The PIT tagging protocol is flexible in that it allows the transponders to be placed in a variety of body locations.
• The protocol has high tag retention rates (>90%) and is non-invasive, since it does not affect fish growth or mortality rates.
• The application of PIT tags can be used to evaluate the success of fishways and other remedial works for supporting crucial life-cycle processes potentially requiring fish passage, such as spawning.

## Graphical abstract

**Figure:**
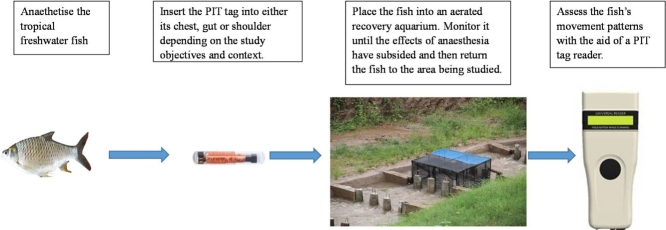

**Specifications Table**

**Table:** 

Subject area	Agricultural and Biological Sciences
More specific subject area	*Fisheries monitoring*
Method name	*Flexible passive integrated transponder*
	*PIT tagging protocols for tropical freshwater species*
Name and reference of original method	B. G. Grieve, L. J. Baumgartner, W. Robinson, L. G. M. Silva, K. Pomorin, G. Thorncraft, N. Ning (2018). Evaluating the placement of PIT tags in tropical river fishes: a case study involving two Mekong River species. *Fisheries Research*. 200: 43–48.

## Method details

### Background

**Fig. 1:**
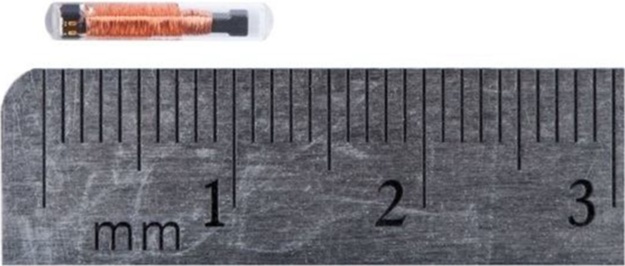
A Biomark HPT12 (12.5 mm) PIT tag (image courtesy of Biomark, Boise, Idaho, USA).

PIT tagging emerged in the 1980s as a novel mark-recapture method for tracking the movements of fish to better understand their ecology [1,2]. The tags consist of small glass capsules, which enclose a uniquely-coded microchip surrounded by a copper wire coil (Fig. 1). Upon being exposed to an electromagnetic field, the copper coil powers the microchip, which then transmits a distinctive signal that can be detected by a low frequency antenna. The technology is effective because the tags are inexpensive, do not need a battery, and can be used on both small- and large-bodied fishes to provide detailed information on their movement patterns [3]. Nevertheless, the technology requires tagged fish to swim through an antenna that emits an electromagnetic field. Consequently, PIT tags are best applied in situations where fish will move past a known location where an electromagnetic transmitting device can be set up, such as in fish migration facilities or near spawning grounds [4].

PIT tagging has been highly successful for increasing the understanding of key migratory temperate freshwater and marine fish species, such as salmonids [5], but its applicability to tropical freshwater species remains largely untested [1]. Nonetheless, tropical river systems are being placed under increasing pressure for their relatively plentiful water resources in response to the continually growing global demand for water. As a stark example of the scale of this issue, there are 11 dams proposed for development on the main stem of the Mekong River over the next decade, and many more have been proposed for its tributary systems [6]. Consequently, there is a critical need for efficient tagging technologies to understand and manage the impacts of river infrastructure developments on the movement ecology of tropical freshwater fishes [21]. In addition to being efficient, these tagging technologies need to be flexible and non-invasive so that they offer the potential to operate under a range of scenarios and/or to meet differing monitoring objectives. Tag placement, in particular, should ideally be flexible enough to allow for differences in study species and attributes, such as morphology and individual size; study objectives and duration; and environmental conditions (e.g. habitat for tangling) [7].

The current study developed and validated a flexible and non-invasive PIT tagging protocol for tropical freshwater fishes, using two Mekong River species – Striped catfish (*Pangasianodon hypophthalmus*) and Goldfin tinfoil barb (*Hypsibarbus malcolmi*) – as test cases. Striped catfish are pangasiid catfishes, which annually migrate to access spawning sites in several upstream locations on the Lower Mekong River [8], while Goldfin tinfoil barb are tropical cyprinids, that form an economically important component of the annual catch of artisanal fisheries from the Mekong [9,10]. Both species were regarded as representative Mekong River fishes because of their body morphologies being typical of the species that reside in the Lower Mekong Basin ([8,22]); and their social and economic importance in the Mekong system [9,10]. The specific aim of this study was to develop a protocol in which PIT tags could potentially be placed in a variety of body locations within the fishes without having any adverse impacts on their growth or mortality rates.

### Fish body locations for tagging

**Fig. 2:**
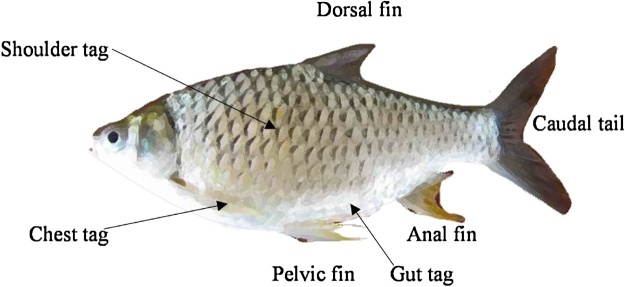
Illustration of a Goldfin tinfoil barb, showing the placement of PIT tags for each PIT tag treatment (chest, gut, shoulder). Fish from the control treatment were not tagged.

Three body locations, with differing advantages, were targeted for this PIT tagging protocol: chest, gut and shoulder (Fig. 2). Chest-located tags are inserted into the pectoral region. Tags in this location have the advantage of being less likely to be accidentally ingested by humans than tags located in body regions that are more sought after for consumption, but they have been shown to have a high shedding rate in some temperate species [11]. Gut-located tags are inserted into the peritoneal cavity (Fig. 2). Tags in this location are even less likely to be accidentally ingested by humans than chest-located tags. They also eliminate the potential for drag forces, and have been shown to have good retention rates in salmonid species [7]. However, they carry a relatively high risk of causing serious welfare issues if the tags are incorrectly implanted [7]. Shoulder-located tags are inserted into the dorsal region, below the dorsal fin (Fig. 2). Tags in this location are easy to apply, and have been shown to have high retention and low mortality rates in temperate species [12]. However, they carry a relatively high risk of being accidentally ingested in this location given that this part of the fish tends to be preferentially consumed by humans. Tag burden impacts were avoided in all body locations by ensuring that the tag-to-body weight ratios were less than 1% (i.e. tags weighed less than 1% of each fish’s body weight in air). Several studies have shown that fish can cope with tag-to-body weight ratios of up to 5% without being adversely impacted [[13], [14], [15], [16]]. Thus, a 1% tag burden limit was considered to be sufficiently conservative to avoid any negative effects on the study fish, and our results ended up supporting this assumption.

### Materials and equipment required for the PIT tagging protocol

• Biomark HPT12 (single-use) needles pre-loaded with PIT tags (12.5 mm 134.2 kHz ISO FDX-B) (Biomark, Boise, Idaho, USA)
• Biomark MK-25 implant gun (Biomark, Boise, Idaho, USA)
• Aqui-S anaesthetic (Aqui-S, Lower Hut, New Zealand)
• Apparatus capable of weighing anaesthetized fish in grams and measuring them in millimetres.

### Fish tagging procedures

Fish were initially surgically anaesthetized using Aqui-S applied at the rate of 25 mg/L [17]. Once anaesthetized (as demonstrated by loss of equilibrium and reduced opercular beat rate), the fish were weighed (g) and measured (mm), and the PIT tags were inserted using a pre-loaded 12.5 mm Biomark needle. Fish were also fin clipped as a record of them receiving PIT tags to allow for potential tag shedding [18]. Fin clipping produces a synthetic extrinsic fish mark that allows for group coding, but has no welfare effects [19]. Once tagging had been completed, fish were placed into recovery aquaria (60 L) containing aerated water, monitored until the effects of anaesthesia had subsided and then returned to their source. This procedure was repeated until all fish were tagged [21].

### Methods validation

We experimentally validated the flexibility and non-invasiveness of the PIT tagging protocol, concurrently on each of the two fish species’ [21]. These experiments involved comparing the growth and survival rates of the tagged fish (i.e. with chest-, gut- and shoulder-located tags) with those of fish that were untagged (i.e. control fish). The experiments were undertaken for each species using four tanks, stocked with 40 fish per tank (N = 160 fish). The four tagging treatments – chest-tagged, gut-tagged, shoulder-tagged and control fish – were randomly, but evenly imposed, such that 10 individuals per tank received each treatment. Each experiment was initiated on 6 June 2016 and run for 50 days, since shedding or tagging-induced mortality rates have previously been shown to be highest during this period [2,20]. The water quality of the tanks was monitored daily, and a 25% water change was undertaken five days per week. Fish were assessed daily for health issues and mortality. Any tags shed were recovered using a magnet, before being scanned using a PIT reader (Biomark, Boise, Idaho, USA), so that they could be related to the fish that had shed the tag. The date, species, initial body location of the tag and tank number were noted. All remaining fish were euthanized using iced water immersion [17] on day 50. Body lengths and end weights of each fish were then assessed, and the fish were dissected to check for tag retention.

We examined the variation among tagging treatments in PIT tag retention (based on the number of tags shed), fish growth (based on change in body weight) and mortality (based on the number of deaths) rates using IBM^©^ SPSS^©^ Version 19 (IBM Corp. Released 2010. IBM SPSS Statistics for Windows, Version 19.0. Armonk, NY: IBM Corp) and the Generalised Linear Models procedure (GENLIN). Overall PIT tag retention rates were found to be greater than 90% for both species, and tag retention rates did not vary among the three tag location treatments (Table 1) (Striped catfish: χ2 = 0.213, df = 3, *p* = 0.975; Goldfin tinfoil barbs: χ2 = 0.26, df = 2, *p* = 0.878). Similarly, neither fish growth (Striped catfish: χ2 = 1.579, df = 2, *p* = 0.454; Goldfin tinfoil barbs: χ2 = 5.735, df = 3, *p* = 0.125), nor mortality rates (Striped catfish: all fish survived the 50-day experiment; Goldfin tinfoil barbs: χ2 = 0.396, df = 2, *p* = 0.82) varied among the tag location and control treatments (Table 1). These results confirm that the PIT tagging protocol is both flexible and non-invasive for Striped catfish and Goldfin tinfoil barbs, and suggest that it could provide an effective technique for quantifying the movements of species in tropical river systems such as the Mekong.

**Table 1:** PIT tag retention, growth and mortality patterns for fish subjected to each treatment. PIT tag retention refers to the proportion of fish still retaining their transponders at the end of the study (and thus control fish were not involved in this assessment); growth is measured as the change in weight over the experiment (in grams); and mortality refers to the proportion of fish that died during the study.

	Goldfin tinfoil barbs			Striped catfish	
	Treatment	Mean	Std. Error	Mean	Std. Error
PIT tag retention	Chest	0.83	0.07	0.85	0.09
	Gut	1.00	0.00	0.98	0.03
	Shoulder	0.93	0.05	1.00	0.00

Growth	Control	53	6.9	119	8.5
	Chest	66	7.4	122	8.9
	Gut	55	6.6	120	8.1
	Shoulder	52	6.8	108	8.5

Mortality	Control	0.00	0.00	0.00	0.00
	Chest	0.10	0.10	0.00	0.00
	Gut	0.15	0.12	0.00	0.00
	Shoulder	0.13	0.09	0.00	0.00
